# Combining structural modeling and deep learning to calculate the *E. coli* protein interactome and functional networks

**DOI:** 10.1038/s41467-026-71166-9

**Published:** 2026-04-11

**Authors:** H. Zhao, C. Velez, A. Naravane, A. Saha, J. Feldman, J. Skolnick, D. Murray, B. Honig

**Affiliations:** 1 Department of Systems Biology, Columbia University Irving Medical Center, 1130 St Nicholas Ave, New York, NY USA; 2 Department of Biochemistry & Molecular Biology, The University of Texas Medical Branch, 301 University Boulevard, Galveston, TX USA; 3 Sealy Center for Structural Biology and Molecular Biophysics, The University of Texas Medical Branch, 301 University Boulevard, Galveston, TX USA; 4 School of Computer Science, Georgia Institute of Technology, 266 Ferst Drive, Atlanta, GA USA; 5 Center for the Study of Systems Biology, School of Biological Sciences, Georgia Institute of Technology, 950 Atlantic Drive, N.W., Atlanta, USA; 6 Department of Biochemistry & Molecular Biophysics, Columbia University Irving Medical Center, 701 W 168th Street, New York, NY USA; 7 Department of Medicine, Columbia University Irving Medical Center, 630 W 168th Street, New York, NY USA; 8 Zuckerman Mind Brain and Behavior Institute, Columbia University, 3227 Broadway, New York, NY USA

**Keywords:** Computational biophysics, Proteomics, Atomic force microscopy

## Abstract

We report on the integration of three methods that predict, on a proteome-wide scale, whether two proteins are likely to form a binary complex. The methods include PrePPI, which uses three-dimensional structure information as a basis for predictions, Topsy-Turvy, which uses a protein language model, and ZEPPI, which uses evolutionary information to evaluate protein-protein interfaces. Testing on the high-quality HINT database of binary PPIs reveals that the integrated method has better performance and identifies more high-confidence interactions than any of the component methods. The AF3Complex algorithm is used to predict the structures of 374 PPIs with a large fraction having at least partially overlapping interfaces with PrePPI models of the same complex. Clustering of the high-confidence *E. coli* interactome yields 385 subnetworks which have high functional coherence. Biological insights derived from the subnetworks, including the annotation of proteins of unknown function, are discussed in detail.

## Abstract (web summary)

We report on the integration of three methods that predict, on a proteome-wide scale, whether two proteins are likely to form a binary complex. Biological insights are derived from subnetworks, including the annotation of proteins of unknown function.

## Introduction

Predicting whether two proteins interact and, where relevant, building a model of the complex they form has become a problem of great interest. This is due both to the multitude of biological applications that the knowledge of protein-protein interactions (PPIs) enables and to major developments in computational approaches, particularly those based on AlphaFold (AF)^1^. The AlphaFold group at Google DeepMind has made models available for over 200 million proteins from multiple organisms^1^ (the AF2 database—AFDB) and, in addition, has developed methods to predict the structures of two or more interacting proteins^2^. The wide adoption of AF-based methods by the biological community has been remarkable, with models of individual proteins from AFDB and of complexes, based on programs such as AlphaFold Multimer^3^ and AF3^2^, becoming common features in the biological literature.

Our interest in this work is the prediction of PPIs on a proteome-wide scale, involving, for example, ~200 million PPIs for human, ~18 million for *S. cerevisiae*, and ~9 million for *E. coli*. The computational cost of carrying out AF-based calculations for so many complexes is prohibitive, especially when accounting for multi-domain proteins, where treating each domain as a separate entity will increase these numbers by over an order of magnitude. Nevertheless, the challenge of deriving proteome-wide interactomes is of great importance as they can reveal the details of complex PPI networks that control cellular function. High-throughput experimental methods, in particular, have made major advances in large-scale PPI detection but have not yet reached the scale of complete interactomes and, further, do not reveal structural information^4,5^.

Computational approaches have also made significant progress on proteome-wide prediction of PPIs, and a wide range of strategies have been deployed^6^. Baker, Cong and colleagues have developed a strategy involving filtering full interactomes using co-evolution-based methods, which significantly reduces the number of PPIs to be considered and then applying structure-informed methods to evaluate the PPIs that remain. In their paper on the *E. coli* interactome^7^, protein-protein docking was used to evaluate the ~22 K PPIs that passed the co-evolution filter, and finally, 804 high-confidence PPIs were identified for further analysis. In more recent studies on the yeast^8^ and human proteomes^9^, putative PPIs were first filtered based on co-evolution after which AF2 was used to identify high-confidence interactions. The endpoint of these studies has been the identification of extremely high-confidence interactions, some of which are not reported in experimental databases and, thus, represent novel interactions. However, such studies have not been implemented to provide proteome-wide PPI interactomes that can then be used to describe functional subnetworks. PrePPI (Predicting Protein-Protein Interactions), which was developed with this goal in mind, adopts a different strategy^10–12^.

The PrePPI pipeline begins with structural models of the individual query proteins and searches for pairs of structurally similar proteins that form a complex in the Protein Data Bank (PDB)^13^. When a PDB complex is found, the query protein models are superimposed on the respective template chains, thus producing a homology model of the query complex. This model is then evaluated with an extremely efficient scoring function, which considers only the quality of the structural alignment and the residues in the query interface that align with those in the template interface. PrePPI’s efficiency is such that it can score billions of models between interacting proteins and their individual domains. PrePPI uses Bayesian statistics to train and score models, and thus, properly chosen training and test sets are of crucial importance. The full version of PrePPI^11^ includes various sources of non-structural evidence, but here we consider only those predictions obtained from the structural modeling component of the pipeline, designated below as SM. Of note, PrePPI has been subject to a number of experimental tests beyond comparisons to various databases. Direct tests have included pulldown experiments^11,14^ and, most recently, the ability to predict proteins that are synthetically lethal to a constitutively activated mutant form of K-Ras in experiments on patient-derived xenograft mouse models^15^.

The quality of PrePPI models depends on the structural similarity of the query proteins to the PDB template proteins. This similarity is reflected in the scoring function and, concomitantly, in the likelihood ratio (LR) associated with a particular PPI prediction. Close homologs will yield good models, while more distant homologs may yield complexes that produce only a weak signal (low LR), which may still be strong enough to provide meaningful evidence for the existence of a PPI. With the goal of further increasing the reliability of PrePPI predictions, we sought a way to integrate the PrePPI pipeline with independent sequence-based methods based on deep learning. This requires that we use a method that, like PrePPI, is fast enough to predict PPIs on a proteome-wide scale. D-SCRIPT^16^ and Topsy-Turvy (TT)^17^, developed in the Berger and Cowen labs, are ideally suited for this purpose.

For a given PPI, we accomplish this integration with a Bayesian approach by multiplying LRs obtained from each source for a given PPI. These sources include: 1) PrePPI structural modeling scores (SM^LR^); 2) ZEPPI Z-scores derived from evolutionary analysis of PPI interfaces with respect to randomly generated interfaces^18^; and 3) TT interaction probabilities, TT^prob^, obtained from a protein language model and network topology considerations^17^. The ZEPPI Z-scores and TT probabilities are transformed into LRs, ZEPPI^LR^ and TT^LR^, through additional training and combined with SM^LR^ into an integrated score, INT^LR^ (see “Methods”).

We report and compare the performance of the four approaches (SM^LR^, ZEPPI^LR^, TT^LR^, and INT^LR^) obtained from training on human PPIs and applied to the *E. coli K12* interactome. We also evaluate the performance of TT^prob^, which was trained on PPIs annotated as physical in the STRING database^19^. We find that PrePPI and TT predict largely different sets of PPIs. The main effect of combining all evidence sources is to identify many more high-confidence PPIs. Of note, TT does not produce atomistic models for complexes, while PrePPI produces models of varying quality dependent on the SM^LR^ associated with the prediction. Both methods can be used to identify PPIs that can potentially be well-modeled with AF-based methods. We chose 374 INT^LR^-predicted PPIs and used the AF3Complex algorithm^20^ to construct models for each. Overall, we find good agreement between the interfaces of AF3Complex and PrePPI models and argue for their combined use in various applications.

Finally, we perform Markov clustering^21^ to identify PPI subnetworks for each of the interactomes. Remarkably, de novo predictions of PPIs based on INT^LR^, TT^LR^, and SM^LR^ produce multiple sets of functionally coherent clusters. We provide an overview of a number of subnetworks from the INT^LR^ interactome and present AF3Complex models for some of the novel PPIs between proteins that are predicted to be cluster members.

## Results

### Performance comparison of different methods

Figure S1 reports Receiver Operator Characteristic  (ROC) curves and Precision Recall (PR) curves for each of the methods − SM^LR^, TT^LR^, ZEPPI^LR^ and INT^LR^ − as evaluated by the 2021 HINT literature-curated high-quality binary database for *E. coli*(denoted here simply as HINT)^22^. At a low false positive rate (FPR), SM^LR^ slightly outperforms INT^LR^, likely due to close structural homologs to PDB structures that contribute to this range, although overall, INT^LR^ performance is superior. Both methods significantly outperform TT^prob^ at low FPR and low Recall. The contribution of ZEPPI^LR^ is most evident at low FPR, where, as has been shown previously, it serves as a useful filter for PrePPI models^16^.

Table 1 reports the area under the ROC curve (AUROC) and the PR curve (AUPR) derived from SM^LR^, TT^prob^, TT^LR^, ZEPPI^LR^, and INT^LR^. TT^prob^ yields values based on the published TT algorithm^17^, which was trained on interactions in STRING defined as physical (STRING-physical)^19^, while TT^LR^ is the result of additional training, also on the STRING-physical database (see “Methods”). TT^LR^ performs slightly better than TT^prob^ when tested on HINT, demonstrating that the additional round of training has not diminished the performance of the published TT algorithm. The results in Table 1 reveal that SM^LR^ exhibits improved performance relative to the two TT-based metrics, but it should be recalled that PrePPI was trained on the HINT database for the human proteome, while both TT methods were trained on STRING-physical, which includes non-direct interactions between proteins in the same multi-protein complex. Finally, INT^LR^ produces the best performance as measured by both AUROC and AUPR, revealing the value of integrating different methods

**Table 1:** Performance of different PPI prediction methods

Method	AUROC	AUPR
SM^LR^	0.88	0.73
TT^prob^	0.85	0.56
TT^LR^	0.88	0.62
ZEPPI^LR^	0.75	0.59
INT^LR^	0.92	0.80

For two FPR ranges (≤ 0.005 and ≤ 0.001), Table 2 reports the total number of PPIs predicted by each method, the number of HINT PPIs recovered, the number of HINT PPIs not predicted (missed), and the number of PPIs not occurring in any experimental database (novel; see “Methods”). Of note, TT^LR^ makes more total predictions than SM^LR^ since there are many cases for which PrePPI cannot find a structural template. ZEPPI^LR^ makes the smallest number of predictions since it depends on paired multiple sequence alignments, which are not available for each PPI. Of the individual methods, SM^LR^ has the highest coverage of HINT, whereas INT^LR^ offers by far the best coverage, highlighting the value of combining methods. Overall, the combination of methods in INT^LR^ yields the largest number of confident predicted PPIs. Further, at an FPR of 0.005, INT^LR^ predicts 60,000 PPIs of which about 48,000 are novel. At an FPR of 0.001, INT^LR^ makes about 19,000 predictions of which about 12,000 are novel. To complement this analysis, Table 3 illustrates that there is limited overlap among predictions of the different methods, which mirrors what has been observed for experimental databases: Different methods identify different sets of PPIs. For this reason, approximating the total number of PPIs in a given organism based on the results from a single method is likely to underestimate the actual number.

**Table 2:** Number of predicted PPIs

# Predicted PPIs (FPR ≤ 0.005)				
	**Total predicted**	**In HINT**	**HINT missed**	**Novel**
SM^LR^	36375	543	1414	26885
TT^prob^	48866	280	1677	40152
TT^LR^	53510	284	1673	43422
ZEPPI^LR^	18263	495	1462	14472
INT^LR^	63896	988	969	47600
**# Predicted PPIs (FPR** ≤ **0.001)**				
**Method**	**Total predicted**	**In HINT**	**HINT missed**	**Novel**
SM^LR^	9724	446	1511	5023
TT^prob^	12023	78	1879	9877
ZEPPI^LR^	5359	321	1636	3894
INT^LR^	19469	739	1218	11718

The top part of the table is for FPR < 0.005, and the bottom is for FPR < 0.001. The headings indicate the set of PPIs referred to in each column.

**Table 3:** Overlap of PPI predictions obtained from different methods

PPI Overlap (FPR ≤ 0.005)				
	**SM**^**LR**^	**TT**^**LR**^	**ZEPPI**^**LR**^	**INT**^**LR**^
**SM**^**LR**^	36375	-	-	-
**TT**^**LR**^	2495	53510	-	-
**ZEPPI**^**LR**^	3730	785	18263	-
**INT**^**LR**^	21761	18431	6917	63896
**PPI Overlap (FPR** ≤ **0.001)**				
	**SM**^**LR**^	**TT**^**LR**^	**ZEPPI**^**LR**^	**INT**^**LR**^
**SM**^**LR**^	9724	-	-	-
**TT**^**LR**^	1266	12023	-	-
**ZEPPI**^**LR**^	1048	375	5359	-
**INT**^**LR**^	7925	4212	2333	19469

Each value is obtained from the ROC curves in Fig. S1.

Given our goal of hypothesis generation, we have used a relatively permissive FPR cutoff ≤ 0.005 for PPIs listed in the online PrePPI database^23^ so as to provide expanded coverage. In the following sections, we focus on higher confidence predictions associated with an FPR ≤ 0.001.

### AF3Complex models of predicted PPIs

In this section, we compare models obtained from PrePPI to AlphaFold predictions. AF3Complex^20^ was used to model 374 PPIs chosen from the set predicted by INT^LR^ with an FPR ≤ 0.001. The subset of 374 PPIs was chosen as described in Methods so as to focus on challenging cases (e.g., <40% sequence identity between the query sequences and their respective chains in the PDB complexes used by PrePPI as modeling templates). AF3Complex uses a predicted interface score, pIS, which focuses on the interfacial residues of the calculated model. The suggested threshold for accurate AF3Complex models is pIS ≥ 0.38, as it was shown that 80% of PDB complexes are recapitulated above this threshold^20^.

**Fig. 1:**
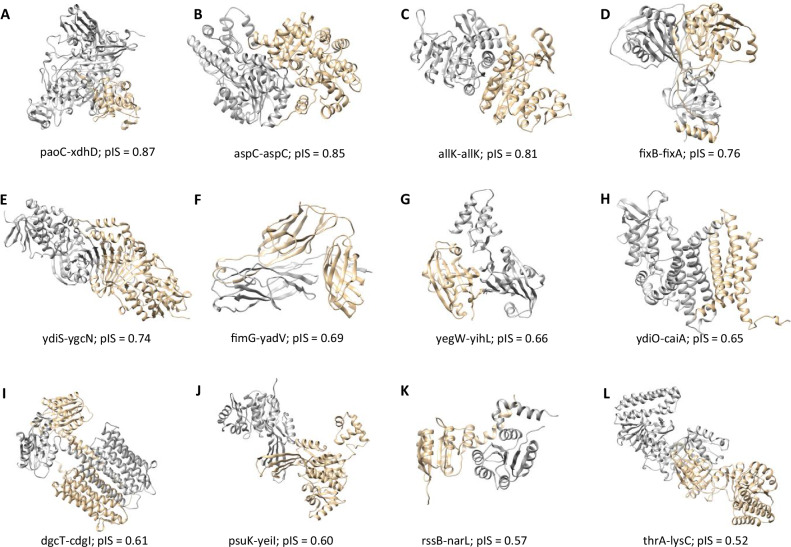
AF3Complex models for high-confidence INT^LR^*E. coli* PPI predictions. Panels **A** through **L** depict AF3Complex models in backbone ribbon representation with one chain colored gray and the other colored gold. The PPIs were chosen from the FPR ≤ 0.001 region of the INT^LR^ ROC curve in Fig. S1 (purple). pIS is the predicted Interface Score from AF3Complex. For simplicity, proteins are denoted by their gene names, and their UniProt IDs are provided in Supplementary Data 1B along with other details related to the predictions. Several models (panels **A**, **F**, **H**, **K**) are discussed in the text.

Table 4 summarizes the results of the comparative analysis of the two sets of models. Of the 374 cases, AF3Complex fails to make a model for 16, and an additional 67 have pIS scores <0.38, which are low confidence predictions, leaving 291 PPIs with pIS scores above this threshold. Consistent with previous studies^24^, we find that complex prediction for homodimers (<pIS> = 0.63) outperforms that for heterodimers (<pIS> = 0.41), and further, all of the cases for which AF3Complex fails are heterodimeric PPIs. Of the 291 PPIs with pIS ≥ 0.38, 164 (56%) have at least 5 interfacial residues on each side in common with the PrePPI model interfaces (some examples, discussed below, are shown in Fig. 1). There are 104 PPIs with more stringent confidence scores (pIS ≥ 0.5; high confidence), and 88 (85%) of these PPIs have interfaces in common with the PrePPI models. These results demonstrate that AF3Complex and PrePPI-SM are likely to produce models with similar interfaces and that the probability of this occurring increases for more confident AF3Complex models. Table 4 also lists the number of novel predictions in each category. It is apparent that a larger fraction of novel predictions is made when pIS ≥0.38 than when pIS ≥ 0.5. This may reflect the observation that AF3-based methods perform less well on PPIs that are less likely to have been included in training, suggesting a lower pIS score for novel PPIs.

**Table 4:** Comparison between AF3Complex- and PrePPI-predicted interfaces

	#PPIs	# novel PPIs
INT^LR^ predictions chosen for AF3complex	374	184
AF3Complex predictions with pIS ≥ 0.38	291	146
Models with pIS ≥ 0.38 and ≥5 IFRs on each side of the interface are common with the PrePPI model	164	74
AF3Complex predictions with pIS ≥ 0.6	104	39
Models with pIS ≥ 0.5 and ≥5 IFRs on each side of the interface are common with PrePPI	88	33

Figure 1 provides a gallery of 12 high-scoring AF3Complex models (pIS ≥ 0.5) for predictions from the FPR ≤ 0.001 region of the INT^LR^ ROC curve (Supplementary Data 1B). Figure S2 contains the models in Fig. 1 structurally superimposed with the respective PrePPI-SM models. In half of the cases, the RMSD between the AF3Complex and PrePPI models is <4 Å, and in most (67%), the RMSD is <6 Å. However, in all cases, interfacial residues are conserved on both sides of the interface (last two columns of Supplementary Data 1B). In Fig. S2 panels F, I, J, and K, the large RMSD values are driven by changes in the domain orientation of the individual partners. Overall, these results highlight the similarity between high-confidence PrePPI and AF3Complex models, particularly in the predicted interface.

The AF3Complex models represent novel PPIs as well as known PPIs for which there is evidence from curated databases. For example, Fig. 1A displays a complex for the observed interaction between PaoC (Aldehyde oxidoreductase molybdenum-binding subunit; gray) and XdhD (Probable hypoxanthine oxidase XdhD; gold). PaoC is involved in purine nucleoside biosynthesis, whereas XdhD participates in cellular detoxification. Both contribute to the synthesis of molybdenum cofactors^25^ which are essential for both pathways. Panel H is the AF3Complex model for the novel PPI between YdiO (Acyl-CoA dehydrogenase; gray) and CaiA (Crotonobetainyl-CoA reductase; gold), which both function in fatty acid oxidation. The models in panels F and K are discussed in the context of functional clusters in the next section.

### Clustered PPIs yield functional subnetworks

At a False Positive Rate (FPR) ≤ 0.001 for the INT^LR^ interactome, there are 19,469 PPIs among 3219 proteins (73% of the *E. coli K12* proteome). The associated network, considering only heterodimeric complexes, is comprised of 18,354 PPIs (edges) among 2913 proteins (nodes). The network was visualized and analyzed in Cytoscape^26^. Network statistics are provided in Supplementary Data 2A. Markov clustering, as implemented in Cytoscape^21^, produced 558 clusters with 2 or more members (Supplementary Data 2B). 139 proteins appear in no clusters, and 124/558 clusters (22%) contain 5 or more proteins, with the largest cluster comprised of 124 proteins (Supplementary Data 2C).

**Fig. 2:**
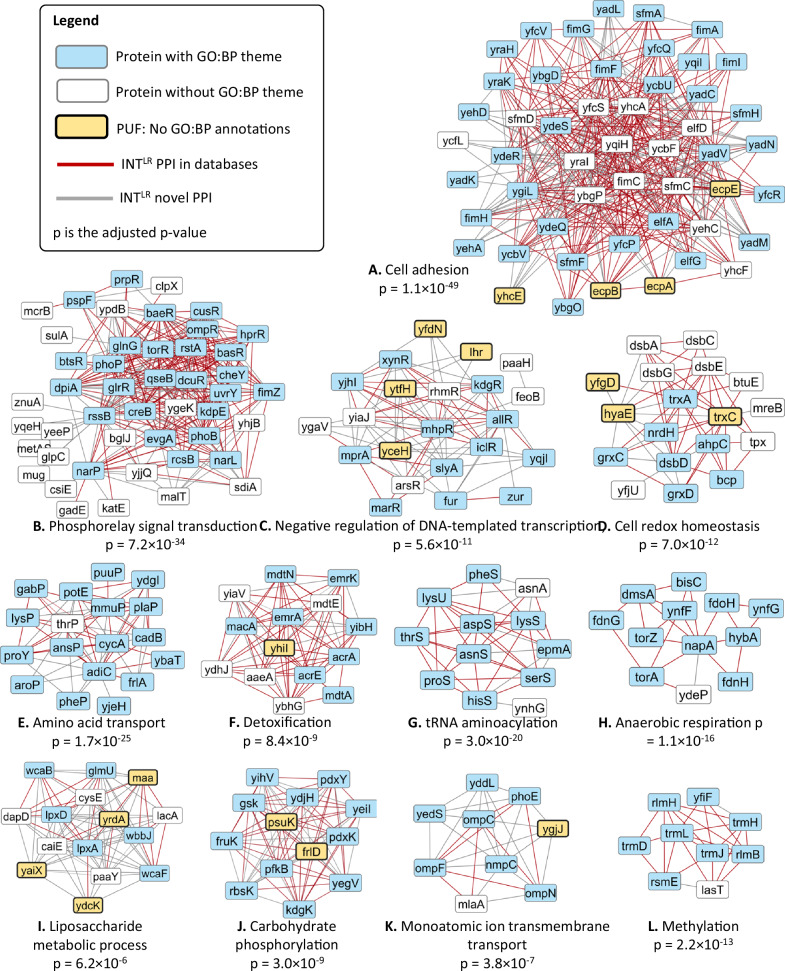
Subnetworks of the Integrated (INT) *E. coli* interactome. The network of INT^LR^ predictions (FPR ≤ 0.001) was clustered^21,26^ and annotated with Gene Ontology (GO) terms^28^. Panels A-L depict subnetworks comprised of a given cluster’s proteins (nodes) and interactions (edges) from INT^LR^ and include the cluster’s functional theme and adjusted *p*-value.

Enriched Gene Ontology (GO)^27^ terms for BP (biological process), MF (molecular function), and CC (cellular compartment) were calculated for each cluster with clusterProfiler^28^ (Supplementary Data 2D). The annotation reveals that the clusters have high functional coherence (Fig. 2). It is important to emphasize that the functional clusters are derived entirely from our predicted INT^LR^ interactome, which contains no explicit functional information. The spectrum of discovered biological processes is broad, encompassing terms including cell adhesion, regulation of DNA-templated transcription, phosphorelay signaling, anaerobic respiration, defense mechanisms, and a wide variety of transport processes and cellular component syntheses. 11,977 of the 18,354 edges (65%) are preserved in the clustered network. 485/558 clusters (87%) are represented by at least one type of annotation (BP, MF, CC), and 385 clusters (69%) are annotated with GO:BP terms (Supplementary Data 2E). The 385 GO:BP clusters contain 257 proteins that are not assigned GO:BP terms in the UniProt Knowledgebase (UniProtKB)^29^ (Supplementary Data 2B), and we define these as proteins of unknown function (PUFs). The occurrence of a PUF in an annotated cluster suggests that the protein may participate in the same pathways and cellular processes in which the cluster is enriched, thus providing predicted annotations. While the SM^LR^ and TT^prob^ interactomes (FPR ≤ 0.001) can be similarly clustered, the SM^LR^ clusters provide denser subnetworks and more statistically significant function annotations (akin to INT^LR^ clustering) than the TT^prob^ clusters.

Among the clusters with no statistically significant function annotation, most (65) have three or two members. While annotations are found for many small clusters, statistical limitations become more pronounced with fewer proteins^30^. The largest unannotated cluster contains 11 members; however, this is an extremely sparse network: Of the 11 edges, 10 are mediated by a single protein, which has only one associated GO biological process term, “polyphosphate biosynthetic process”.

Supplementary Data 2E contains the most statistically significant GO:BP terms for the 117 annotated clusters with five or more members. Examples of annotated clusters and their subnetworks, some containing PUFs, are provided in Fig. 2. For simplicity, proteins are denoted by their gene names and identified as necessary below. Those that are formally annotated with the GO:BP term indicated are colored blue. The remaining proteins (white and orange nodes) are hypothesized to contribute to the annotated function since interacting proteins are more likely to share the same functions and biological processes. In the following, we elaborate on four clusters (Fig. 2A–D). Red edges represent predictions by INT^LR^ that appear in databases, and gray edges represent novel predictions (Supplementary Data 2 F). We also highlight predicted annotations for PUFs (orange nodes) and provide AF3Complex models for a couple of novel PPIs to demonstrate how our clusters are a source of functional annotation and hypothesis generation.

Figure 2A depicts a subnetwork highly enriched in proteins involved in “cell adhesion,” with 33 of the 50 proteins assigned this GO:BP term, suggesting that the remaining 17 may also participate. Indeed, 11 of the 17 proteins have the annotation “cell wall organization,” a related biological process that involves cell surface interactions. Of the four PUFs, yhcE (the UPF0056 membrane protein YhcE) is a plasma membrane-associated transmembrane protein that interacts with five of the proteins assigned to “cell wall organization” (fimC, yfcS, ybqP, yraI, and yqiH). Since the last steps of cell wall synthesis occur at the plasma membrane^31^, we predict that yhcE may also participate in adhesion. Further, the other three PUFs, ecpA (Common pilus major fimbrillin subunit EcpA), ecpB (Probable fimbrial chaperone EcpB), and ecpE (Probable fimbrial chaperone EcpE), are part of the operon that encodes the pilus, the hair-like cell-surface appendage that mediates cell adhesion^32^, so that we predict they also contribution to cell adhesion. Finally, Fig. 1F depicts the AF3Complex model (pIS = 0.69) for the novel interaction between two known cell adhesion proteins: the fimG (fimbrial adhesion protein fimG) and yadV (Probable fimbrial chaperone YadV), a chaperone for fimbriae assembly at the pilus^33^. yadV facilitates the folding of fimG, and the model provides a structural mechanism for this process.

Figure 2B is the subnetwork for the “phosphorelay signal transduction system.” Phosphorelays are two-component “intracellular signal transduction” systems that respond to environmental changes through a series of phosphorylation events involving histidine kinases and response regulators. One-third of the proteins in this cluster are part of a “protein-DNA complex” (GO:CC), including rssB (Regulator of RpoS), which directs the expression of genes involved in survival under stressful conditions, and narL (Nitrate/nitrite response regulator protein NarL), a transcription factor that regulates the expression of genes involved in anaerobic respiration. Figure 1K depicts the AF3Complex model (pIS = 0.57) for the novel PPI between rssB (gray) and narL (gold), which is mediated by the proteins’ receiver domains. The model suggests a mechanism for the crosstalk between stress response and anaerobic respiration whereby rssB is recruited to DNA through the interaction of its receiver domain with narL and thus mediates its activity^34^.

The subnetwork in Fig. 2C is involved in “negative regulation of DNA-templated transcription”. The four PUFs—yfdN (Uncharacterized protein YfdN), lhr (Probable ATP-dependent helicase Lhr), ytfH (Uncharacterized HTH-type transcriptional regulator YtfH), and yceH (UPF0502 protein YceH)—are predicted to interact directly with proteins associated with this GO:BP term. In addition, ytfH, which contains a transcriptional regulator domain, is predicted to make novel interactions with two essential proteins (which share a negative genetic interaction) in this cluster: the transcriptional activator mhpR (DNA-binding transcriptional activator MhpR) and the transcriptional repressor allR (HTH-type transcriptional repressor AllR)^35^, further supporting the predicted annotation in this case.

The subnetwork in Fig. 2D has the annotation “cell redox homeostasis.” The PUF trxC (Thioredoxin 2) interacts with trxA (Thioredoxin 1), and both are part of the thioredoxin (Trx) system, which maintains thiol-based redox homeostasis^36^. trxC is also predicted to interact with proteins that are localized to the “periplasmic space” (GO:CC), which is the oxidizing environment for cell redox homeostasis. Although trxC is annotated as a “cytoplasmic/cytosolic protein” (GO:CC), it is predicted to interact with periplasmic proteins. Taken together, these predictions provide strong evidence that trxC plays a role in cell redox homeostasis. The PUF hyaE (Hydrogenase-1 operon protein HyaE), which has oxidoreductase activity (GO:MF), also interacts with periplasmic proteins, suggesting its involvement as well.

Overall, our analysis illustrates how the INT^LR^ interactome and its subnetworks can find novel members for canonical processes, predict annotations for PUFs, and prioritize PPIs for high-resolution structural modeling.

## Discussion

Here, we have explored the combined use of different computational approaches to predict PPIs on a proteome-wide scale. The three methods leverage different data sources. PrePPI-SM leverages structure relationships, ZEPPI filters PrePPI predictions by analyzing the surfaces of PrePPI models, and TT predictions are derived from sequence. As evident from Table 3, there is limited overlap between the different methods. Integration improves overall performance, with the greatest effect being a dramatic increase in interactome coverage, although INT^LR^ also yields the best performances in terms of AUROC and AUPR. Limited overlap is reminiscent of what is observed for experimental methods^10^. Since each method has its own strengths, weaknesses and degrees of uncertainty, simply treating all predictions on equal footing is problematic. We have dealt with this issue by integrating different methods through a Bayesian approach while comparing the performance of individual methods on the same test set. As the number of methods, especially those based on ML/DL, continues to grow, it will be important to find novel ways to compare, contrast and integrate them to extract maximum insight.

In addition to comparisons of PPI prediction methods, we have also compared the 3D models of binary complexes produced by PrePPI-SM and AF3Complex. Of note, PrePPI uses full-length proteins as well as individual domains in its pipeline, while AF3-based methods generally involve the co-folding of full-length proteins. We find that there is good agreement between the interfacial regions predicted by both methods, with the level of agreement improving for more confident AF3Complex models (all PrePPI models are associated with a high confidence INT^LR^ FPR cutoff of ≤ 0.001). Since both methods rely on PDB templates, either through structure alignment or through machine learning, it is not surprising that overall agreement is good. PrePPI’s advantage is that it can be easily applied to explore all pairwise combinations of proteins in a proteome, while AF3-based methods are too slow for this purpose. PrePPI alone as well as TT and other fast PPI predictions can be used as initial screens for downstream AF3 applications, but even when computational demands are not an issue, it is worth using both AF3 models and PrePPI models in focused applications. Neither method is guaranteed to produce accurate PPI models, and, indeed, AF3Complex fails to produce models for 16 out of the 374 cases for which PrePPI provides a high-confidence model and produces low-confidence models for another 67 (Supplementary Data 1).

As summarized in the Introduction, Baker, Cong and coworkers have established a computational pipeline that delivers a small number of very high confidence predictions for *E. coli*^7^. This general strategy has been maintained in recent studies of the human interactome^9^ although many features of the pipeline have changed as required by the challenge of the much larger human proteome. For *E. coli*, Cong et al. report 804 de novo predictions without additional input from experimental databases. In contrast, our approach is to report a much larger number of predicted PPIs with the goal of obtaining a more global perspective of an interactome. For example, INT^LR^ makes 19,469 predictions at an FPR cutoff of 0.001. This number is based on a more permissive cutoff than used by Cong et al., but one that has produced predictions that have been validated in multiple applications^12,14,15^. Where the line is drawn, of course, depends on one’s goals.

In order to leverage the *E. coli* interactome reported here, we clustered the interactome based on the 19,469 INT^LR^-predicted PPIs (FPR ≤ 0.001). Although no functional information was incorporated into our PPI predictions or their clustering, the subnetworks generated in this way were found to have functional coherence as indicated by the Gene Ontology biological process (GO:BP) terms statistically overrepresented for most clusters^27,28^. This finding offers strong support for the relevance of the interactome provided by our PPI predictions.

Interactome clustering offers a unique proteome-wide perspective of known and novel functional relationships, including the annotation of proteins previously designated as proteins of unknown function (PUFs). Based on Gene Ontology information provided by UniProtKB, we designated 1063 (26%) of the 4402 proteins in the *E. coli* K12 reference proteome as PUFs. 433 appear in the PPIs that comprise the FPR ≤ 0.001 interactome. Because of their placement in functionally annotated clusters, we predict GO:BP annotations for 257 (60%). This level of annotation is due to our systematic and unbiased method for PPI prediction and our extensive coverage of the interactome space.

In summary, we have integrated different computational methods that are efficient enough to provide PPI predictions for entire interactomes. Further, PrePPI-SM is based on structural models for thousands of binary PPIs, and many of these appear to be similar to those obtained from AF3Complex. These predictions are contained in the PrePPI website^23^, which also enables interactive interrogation of functional clusters. We anticipate that the combined structure/function-based description of full interactomes will prove extremely useful in multiple biomedical applications.

## Methods

All calculations were performed for the 4,402 *E. coli K12* (taxonomy identifier 83333) proteins from the UniProtKB reference proteome with identifier UP000000625^37^.

### PrePPI SM^LR^

The PrePPI algorithm has been described multiple times in the literature^11,12,14^, and its main features are summarized above. Although PrePPI incorporates many features that enable the prediction of PPIs that do not involve direct physical interactions, here we focus on the structural modeling component, SM, which predicts the physical interaction of two monomeric structured domains. PrePPI’s rapid SM scoring function allows us to evaluate billions of domain-domain and full-length protein PPI interactions. Bayesian statistics are used to calculate a likelihood ratio, SM^LR^, for a given putative PPI. This requires the use of true positive (TP) and true negative (TN) datasets. The most recent version of SM^LR^ was trained on the human HINT literature-curated high-quality binary database^22^ as the TP set and any PPI not functionally annotated in a large number of experimentally derived databases as the TN set^10^. Out of the 9.6 million potential protein-protein pairwise combinations in *E. coli*, templates cannot be found for 4 million, while PrePPI is able to build models and evaluate the LR for 5.6 million. Of these, the 36,375 PPIs associated with an FPR ≤ 0.005 appear in the online PrePPI database^23^ for *E. coli*, but all entries are available for download.

### ZEPPI^LR^

ZEPPI^18^ leverages co-evolution and conservation signals across protein-protein interfacial contacts to compute a Z-score based on comparisons to randomly constructed interfaces derived from residue pairs found anywhere on a protein surface. ZEPPI relies on species-matched paired multiple sequence alignments, which are compiled using Jackhmmer 2.0^38^ to search for homologous sequences for each protein in the EggNog 5.0 database^39^. In all, we were able to calculate effective ZEPPI scores for 5 M PPIs. ZEPPI can be used to evaluate any complex whose atomic coordinates are known, but here we calculate ZEPPI scores for PrePPI models. In order to incorporate ZEPPI into a Bayesian network, we converted ZEPPI Z-values into LRs defined here as ZEPPI^LR^. This is accomplished by training ZEPPI Z-scores on the HINT human dataset and then using the trained model on *E. coli*.

### Topsy-Turvy

Topsy-Turvy^17^ (TT) is a sequence-based method to predict PPIs from the Berger/Cowan labs. TT was developed following the earlier introduction of D-SCRIPT^16^, which is based on a protein language model. TT also leverages global “top-down” methods that infer properties from patterns of known PPIs. We applied TT to all *E. coli* sequences, resulting in 9.5 million predicted PPIs. Like PrePPI, TT is extremely fast and can be applied proteome-wide. It outputs interaction probabilities, which, for integration with PrePPI, must be converted to LRs. TT was trained on the STRING^19^ physical database for human PPIs. We converted PPI probabilities into LRs by training on the same data set. We define TT^prob^ as containing interaction probabilities obtained from the published version of TT, while TT^LR^ provides LRs from our additional stage of training.

### Bayesian model

TT^LR^ was combined with SM^LR^ of PrePPI and ZEPPI^LR^ to yield INT^LR^, which integrates all three evidence sources used in this work. The three LR scores were trained on human data and then combined and evaluated on *E. coli* using a naïve-Bayesian model, which is a general, statistics-based machine-learning framework (Eq. (1)). The likelihood ratio for feature *j* is defined as the probability of observing feature *j* in the true-positive (TP) set divided by that in the true-negative (TN) set (Eq. (2)). The positive and negative ratio used for the training was 1:1000 as this ratio is close to the estimated true and false PPI ratio in *E. coli*. For feature *j* in the bin *k* of class *c*, a Laplace smoothing function with pseudocount is introduced to ensure that the smoothed probability is close to 0 in the zero-count case with no denominator error and does not cause much disturbance to other cases (Eq. (3)). In Eq. (3), *K*_*j*_ is the number of bins for feature *j, α* is the pseudocount set equal to 1. The associated code can be found in our GitHub repository as described in Data Availability.

1 $${{INT}}^{{LR}}={{SM}}^{{LR}}\cdot {{ZEPPI}}^{{LR}}\cdot {{TT}}^{{LR}}\,$$

2 $${{LR}}_{j}=\frac{P(j|{TP})}{P(j|{TN})}$$

3 $$P\left(j | c\right)=\frac{{{Count}}_{c,j,k}+{{\alpha }}}{N+{{\alpha }}{K}_{j}}$$

During training, ZEPPI scores and TT probabilities are divided into bins using Doane’s formula for automatic binning^40^. 38 bins are used for ZEPPI and 38 for TT. Doane’s formula adjusts the bin count based on skewness. This makes it more suitable for data with a non-normal distribution and large sample sizes. Since there are 5.6 million, 5 million and 9.6 million predicted PPIs for SM^LR^, ZEPPI^LR^ and TT^LR^, respectively, there will be many PPIs for which no value can be calculated for SM^LR^ and/or ZEPPI^LR^. In these cases, the corresponding LRs are set to 1 when ROC and PR curves are calculated for INT^LR^. Thus, a large fraction of our calculated LRs is based entirely on TT.

### Choice of PPIs to be studied with AF3Complex

INT^LR^ predictions were chosen for modeling by AF3Complex based on the following filtering procedure. Starting with the 19,469 protein-protein interactions with INT^LR^ values below the FPR cutoff of ≤ 0.001 (Table 1), PrePPI-SM models were constructed by superimposing models for the query proteins onto the respective template chains in PDB complex structures. We selected PPIs for which the PrePPI-SM models have at least 5 interfacial contacts between residues whose heavy atoms satisfy a distance cutoff of <6 Å. From this set, we retained challenging cases involving pairs whose members have low local sequence identity (<40%) and high alignment length (>100 residues) with the corresponding chains from the PDB template complex selected by PrePPI for model evaluation. Further, to avoid overrepresentation, no individual protein appears more than six times in this set. Finally, from the highest-confidence PPIs matching these criteria, 374 were chosen so that half appear in databases and, thus, the other half are novel predictions. Detailed information on the final set of 374 PPIs is provided in Supplementary Data 1A. Note that all 374 PPIs are confident predictions in the sense that the INT^LR^ is always high (FPR ≤ 0.001).

### Interactome clustering and function annotation

The INT^LR^ interactome was visualized and clustered in Cytoscape version 3.10.4^26^. The Markov clustering algorithm, as implemented in Cytoscape^21,26^, was used to cluster the network obtained from the INT^LR^ PPI predictions with FPR ≤ 0.001. Default parameters were used, and all edge weights were set to 1. Over-Representation Analysis for Gene Ontology (GO) terms (biological process (BP), cellular compartment (CC), and molecular function (MF)) was performed with the enrichGO function in the clusterProfiler R package (v4.14.6)^28^. The org.EcK12.eg.db R package^41^ was used for *E. coli strain K12* genome-wide annotation with gene symbols. Enriched GO terms with an adjusted *p*-value ≤ 0.05 were retained. The adjusted p-value was determined with the Benjamini-Hochberg (BH) method to correct for multiple hypothesis testing. Network, clustering, and annotation results are provided in Supplementary Data 2.

### Reporting summary

Further information on research design is available in the Nature Portfolio Reporting Summary linked to this article.

## Supplementary information

Supplementary Information

Description of Additional Supplementary Files

Supplementary Data 1

Supplementary Data 2

Reporting Summary

Transparent Peer Review file

## Data Availability

All predictions generated in this study, including genome-wide PPI predictions for human and *E. coli* using three different methods (PrePPI, ZEPPI, and D-Script-TT), as well as the integrated predictions derived from the Bayesian model, have been uploaded to Figshare [10.6084/m9.figshare.31362145]. The PrePPI predictions can also be downloaded from the PrePPI website [https://honigcomplab.c2b2.columbia.edu/PrePPI]. Supplementary Tables S1, S2 are available. The source data underlying Supplementary Fig. S1 is provided as a Source Data file on Github repository [https://github.com/honig-lab/BayesianModel-for-Ecoli-PPI/tree/main/data].

## Code availability

The code and tutorial for integrating PrePPI, ZEPPI, and D-Script-TT inputs through a Bayesian framework are available on the GitHub repository [https://github.com/honig-lab/BayesianModel-for-Ecoli-PPI] and from Zenodo [10.5281/zenodo.18684873].
