# Supplementary material for: Combining structural modeling and deep learning to calculate the E. coli protein interactome and functional networks
Source: Nat Commun. 2026 Apr 11;17:5093. doi: 10.1038/s41467-026-71166-9 (PMC13246756; doi:10.1038/s41467-026-71166-9)

Figure S1. Evaluation of PPI prediction methods. A. Receiver Operating Characteristic (ROC) curve and B. Precision Recall (PR) curve as tested on the HINT high-quality literature-curated binary *E. coli* PPI set for SM^{LR} (red), $ZEPPI^{LR}$ (green), TT^{Prob} (blue), TT^{LR} (orange), INT^{LR} (purple), and random performance (gray).

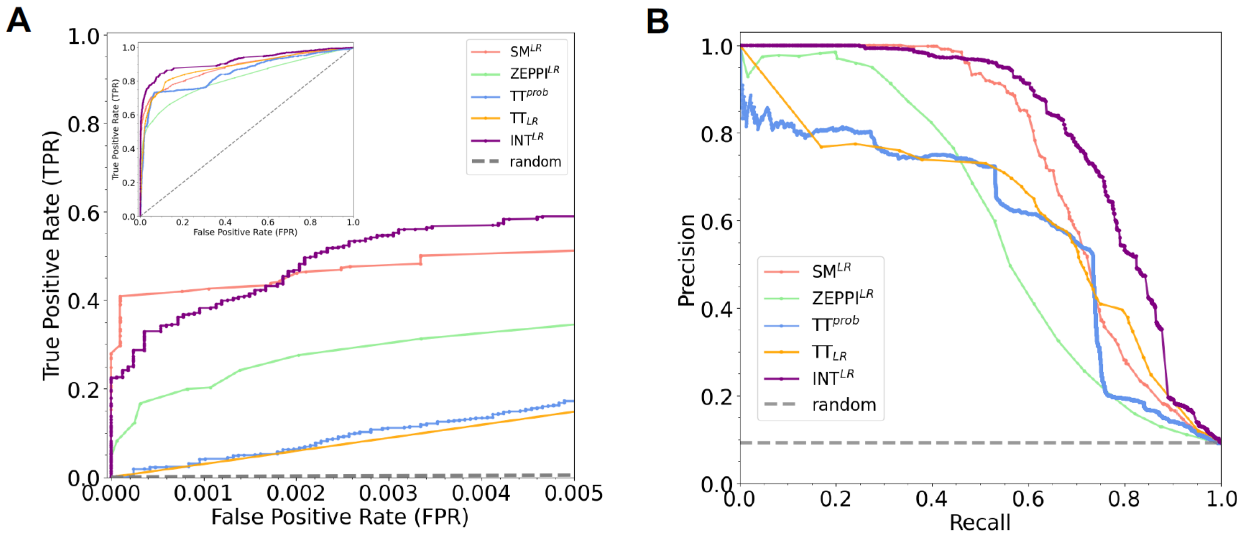

Figure S2. Panels A-L provide the superposition of PrePPI and AF3Complex models. Models are represented in backbone ribbon representation. The same AF3Complex models from Figure 2 are depicted (gray and gold) superimposed with the PrePPI models for the same PPI (cyan and brown). Proteins are denoted by their gene names; pIS is the predicted interface score for the AF3Complex models; and RMSD is from the superposition of the AF3Complex and PrePPI models. Information on the superpositions is provided in Table S1B.

	AF3Complex	PrePPI
Protein 1		
Protein 2		

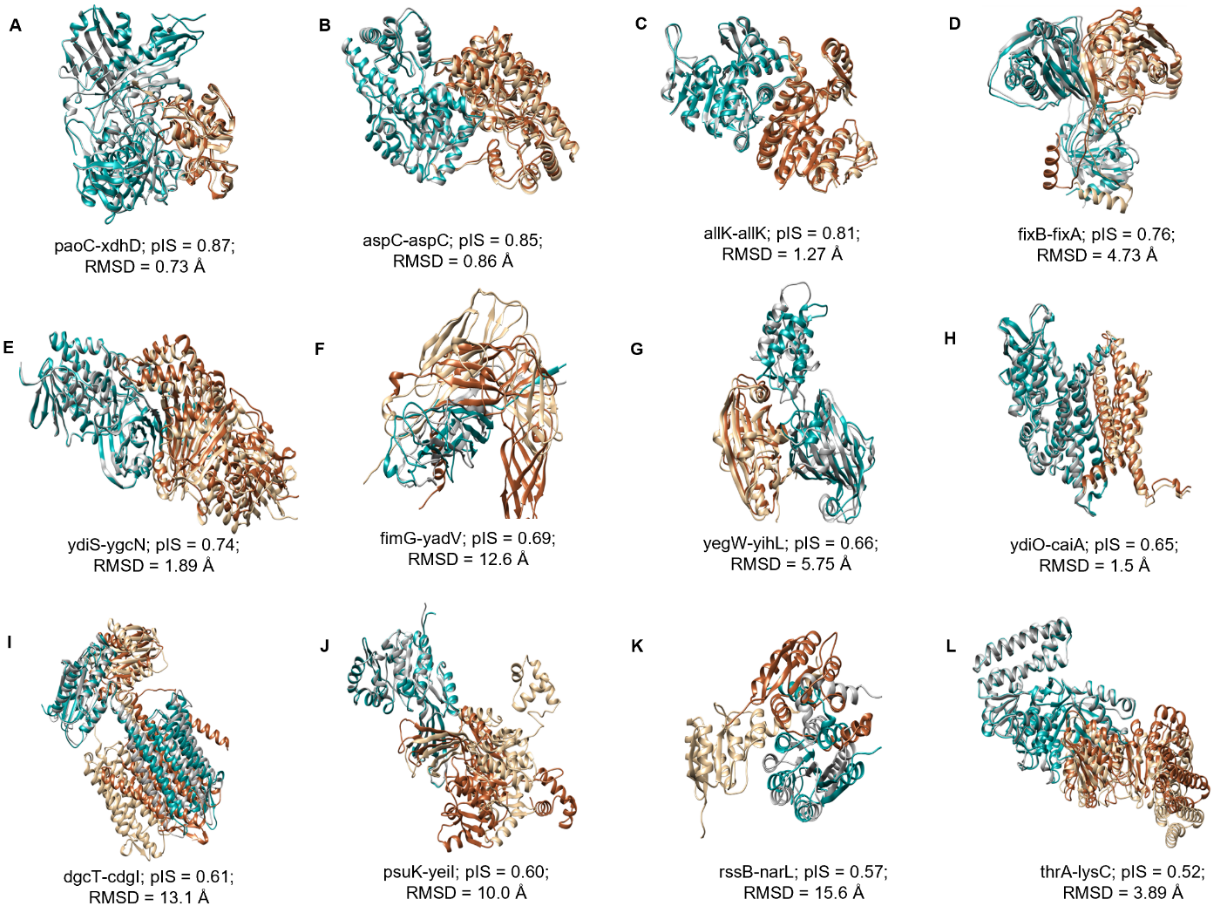

Supplement: Supplementary file 1 — Supplementary Information [file 41467_2026_71166_MOESM1_ESM.pdf]
