# Supplementary material for: Combining structural modeling and deep learning to calculate the E. coli protein interactome and functional networks
Source: Nat Commun. 2026 Apr 11;17:5093. doi: 10.1038/s41467-026-71166-9 (PMC13246756; doi:10.1038/s41467-026-71166-9)
Supplement: Supplementary file 2 — Description of Additional Supplementary Files [file 41467_2026_71166_MOESM2_ESM.pdf]

Description of Additional Supplementary Files

File Name: Supplementary Data 1

Description: Contains 374 selected PPIs predicted with high-confidence integrated likelihood ratio (LR). These interactions are considered challenging because the protein pairs exhibit low local sequence identity. Detailed selection criteria are described in the *Methods* section of the paper.

File Name: Supplementary Data 2

Description: Contains network construction, clustering, and functional annotation results. Derived from interactome clustering analysis.
