# Supplementary material for: Combining structural modeling and deep learning to calculate the E. coli protein interactome and functional networks
Source: Nat Commun. 2026 Apr 11;17:5093. doi: 10.1038/s41467-026-71166-9 (PMC13246756; doi:10.1038/s41467-026-71166-9)
Supplement: Supplementary file 6 — Transparent Peer Review file [file 41467_2026_71166_MOESM6_ESM.pdf]

nature portfolio

Peer Review File

Combining structural modeling and deep learning to calculate the *E. coli* protein interactome and functional networks

Corresponding Author: Professor Barry Honig

This file contains all reviewer reports in order by version, followed by all author rebuttals in order by version.

Version 1:

Reviewer comments:

Reviewer #1

(Remarks to the Author)
Prediction and structural characterization of protein-protein interactions is a key problem in molecular biology. There has been great progress in this direction based on application of deep learning techniques (AlphaFold and other approaches). However, for large interactomes these techniques generally are computationally expensive. Computationally efficient approaches based on structural and functional similarity are important for structural and functional analysis of proteome-wide systems.

The authors present a detailed account of a comprehensive study combining complementary approaches to structural and evolutionary characterization with deep learning techniques. The proteome-wide analysis resulted in high-quality prediction of protein-protein interacting pairs, and accurate structural characterization of the interaction. Importantly, it also produced meaningful functional clustering of the interacting proteins.

The approaches were appropriately benchmarked on the available data. The study addresses an important biological problem. The paper is well written and illustrated. As such it should be of great interest to the biological community.

Reviewer #3

(Remarks to the Author)
The manuscript "Combining structural modeling and deep learning to calculate the E. coli protein interactome and functional networks" describes the use of a combination of tools for the prediction of proteome-wide protein-protein interactions in E. Coli.
This approach allowed to identify potential novel interactions, predicted with high likelihood.

By clustering the obtained pairings, the authors managed to identify subnetorks and describe biological process associated to them, thus allowing to predict the potential function of unannotated proteins within the network.
I think that the described method is of great interest to annotate potential grey zones in the annotation of proteins / biological assemblies.

I was surprised not to see any code / script / repository associated with the described method. I unfortunately cannot apply it on my own system without having to write it myself from scratch.

Here are some remarks related to the text:

l.174: It is not clear why only SM-lr, TT-prob, TT-lr and Int-lr are evaluated against the HINT E.Coli binary dataset, but not ZEPPI-lr. Is it because it requires structural model to perform predictions ? But because in the introduction is is mentioned that Int-lr contains prediction from ZEPPI-lr, therefore I am getting confused. I would suggest adding the reasons why ZEPPI-lr is not part of Table 1, nor in both Figure 1 and 2, or to add it. Maybe a short description of what is required by ZEPPI in the text would help? And in l.210, it is again stated that ZEPPI-lr is part of the Int-lr, making it again surprisingly confusing.

l.192: AU, potentially meaning Area Under, is not defined.

l.196: In figure 2, I am surprised not to see any comparisons against the HINT E.Coli dataset. How many predictions are corrected, how many are missing, how many new potential binary pairings have been found?

l.207: I have the feeling that I need more explanations about the content of those 400 PPI chosen. How many of them are already reported in the literature? How many of them are new ones?

l.214: to my understanding, I would guess that the actual sentence should be "… 80% of PDB complexes are recapitulated ABOVE this threshold", instead of "… 80% of PDB complexes are recapitulated at this threshold"?

l.215: I would suggest to add reasons why AF3Complex is not producing scores for the 18 entries.

l.214-215 and Table S1A: I see that there is a substantial amount of homo-dimeric complexes that are selected among the 400 PPI chosen. I would suggest to discuss this behavior and the underlying number of homo- and hetero-mers that are modelled. Is there a bias in the selection methods where homo-dimeric complexes can easily be chosen as the < 40% cutoff on sequence identity can only be met on the same chain and not on both ?

l.232-232: For well scored complexes, if both PrePPI (homology modelling method) and AF3Complex (machine-learning method) produce similar results, I have the feeling that there might be a bias in the use of pIS score. Indeed AF3Complex has (most probably) seen the complexes used by PrePPI to model the complex and therefore these complex are not so novel. My concerns is more related to the possibility for the overall pipeline (Int-lr into AF3Complex) to validate new / undiscovered pairings.

l.263: If I do understand well, for the remaining 73 clusters (558 - 485), not any of the proteins are annotated ? Are these big clusters ? or simply pairings of unannotated proteins ?

l.731: Figure 4. I would suggest to add the number of novel PPI and known PPI from curated databases, as counting the red and grey lines is hard (impossible). Possibly as percentages of known and unknown ? and reporting the total number of edges present in each cluster ?

l.302: AF3Complex suddenly becomes AlphaF3Complex ?

l.327-376: Discussion: In my opinion, this resembles more a conclusion rather than an actual discussion. Therefore the discussion is missing from the current version of the paper.

l. 394: It is unclear why 4 million predictions cannot be made by PrePPI.

l.408-409: The ZEPPI-lr is trained on the HINT E.Coli but not human, making it the only method trained on E.Coli and also used to predict E.Coli partners ? Why would it be ? And not trained on Human HINT instead ?

l.430: "During training, …": it is not clear here what is the training set and the loss function use for the Bayesian model.

Version 2:

Reviewer comments:

Reviewer #1

(Remarks to the Author)
The revised manuscript in its current form is suitable for publication.

(Remarks on code availability)

Reviewer #2

(Remarks to the Author)
The revised paper provides fast pipeline to identify protein complexes at genomic scale demonstrating its application on Ecoli-PPI, which is current frontier in molecular systems biology. It uses template based pipeline PrePPI, assessor of interface quality ZePPI, and STRING trained sequence based prediction and uses combined model to predict interactions. The model is fast for use at proteomic scale prediction . It gives comparable results to AF based models, and also can be used in conjunction. The authors demonstrate that clustering proteins based on predicted PPIs can provide functional annotation. Overall the authors present blueprint for fast analysis of organism PPI networks and provide predictions for EColi. The papers is well written, methodology is well described.

(Remarks on code availability)
The instructions appear adequate and there are test examples related to the paper.

Reviewer #3

(Remarks to the Author)
I appreciated the point by point responses to the reviewers and believe that the manuscript has accordingly improved in the right direction.
Nevertheless, I am allowing to add several issues and remarks that I could find that could optimize once more that text:

l.199: Small typo where “At an FPR…” should be (in my opinion) “At a FPR…”

l.200: same

l.221: I do not fully agree with the sentence “Thus, we regard the 83 protein complexes as not being predicted by AF3Complex.”. In my opinion, the predictions are made, but not trustworthy, “thus, we considered the 83 protein complexes poorly predicted by AF3Complexe as false positive” ?

l. 207: General comments about the “AF3Complex models of predicted PPIs”, in relation to the following part “Clustered PPIs yield functional subnetworks”. I have the feeling that because of the stringent selection of the 374 diverse binary PPIs, some of the failures in modelling could come for the fact that predicted PPIs are belonging to higher order macro-molecular complexes (or with a different stoichiometry). Have you considered to model not only the predicted pair, but its full subnetwork ? And, in the same thought, are those 83 poorly predicted binary complexes coming from predictions made by TTlr only ?

l.283: small typo, missing the verb in “however this an extremely sparse network” that should probably become “however this is an extremely sparse network”

l.345: maybe small typo in “The three methods leverage differ data sources.” that should probably become “The three methods leverage different data sources.”

l.348: small typo, the parenthesis should become a coma

l.349-350: I would modify “…yields the best results for AUROC and AUPR” to “…yields the best performances in terms of AUROC and AUPR”

l.377: et al. should be in italic.

l.378: I feel strange the “…is to report…”. Shouldn’t it be “…reports…” or “… is reporting…” ?

l.381: et al. should be in italic.

Table 1: I would add the number of undetected PPI from HINT E.Coli in all cases, even though this is partially present in the PR-AUC, and in the text (l.192 to 196)

Table 2: I feel there is a mistake in the caption that mention TTprob while the table contains the values for TTlr. Also, I would display only half of the table, as it is a symmetric one, and therefore add some unnecessary noise. Finally, why not also showing the values at FPR<000.1 ?

(Remarks on code availability)
The README.md is missing the python3 version required.

It is missing instructions on how to write the UniprotID_pair. Is it coma separated ? is it underscore separated ?

What is the filename that should be given to this file ?

In both `Train_on_human.ipynb` and `Train_on_human.ipynb, `localpath_prefix` variable must be changed. I would suggest to add a comment before to make sure the user will do it.
Same for `filetoread\d?`: the hardcoded filepaths will likely not match anything present on the user computer and nothing present in the repo.

There is no instructions on how to download the HINT and STRING databases.

I could not find anything related to the Cytoscape analysis.

While the notebooks are showing the basics of the analysis, reproducing it is not possible, as many files cannot be found. Maybe a storage space such as zenodo, or making the OneDrive open could be a solution ?

Open Access This Peer Review File is licensed under a Creative Commons Attribution 4.0 International License, which permits use, sharing, adaptation, distribution and reproduction in any medium or format, as long as you give appropriate credit to the original author(s) and the source, provide a link to the Creative Commons license, and indicate if changes were made.
In cases where reviewers are anonymous, credit should be given to 'Anonymous Referee' and the source.
The images or other third party material in this Peer Review File are included in the article's Creative Commons license, unless indicated otherwise in a credit line to the material. If material is not included in the article's Creative Commons license and your intended use is not permitted by statutory regulation or exceeds the permitted use, you will need to obtain permission directly from the copyright holder.
To view a copy of this license, visit https://creativecommons.org/licenses/by/4.0/

Tables S1 and S2 are now available on Figshare https://dx.doi.org/10.6084/m9.figshare.30822977.

Reviewer #1 (Remarks to the Author):

Prediction and structural characterization of protein-protein interactions is a key problem in molecular biology. There has been great progress in this direction based on application of deep learning techniques (AlphaFold and other approaches). However, for large interactomes these techniques generally are computationally expensive. Computationally efficient approaches based on structural and functional similarity are important for structural and functional analysis of proteome-wide systems.

The authors present a detailed account of a comprehensive study combining complementary approaches to structural and evolutionary characterization with deep learning techniques. The proteome-wide analysis resulted in high-quality prediction of protein-protein interacting pairs, and accurate structural characterization of the interaction. Importantly, it also produced meaningful functional clustering of the interacting proteins.

The approaches were appropriately benchmarked on the available data. The study addresses an important biological problem. The paper is well written and illustrated. As such it should be of great interest to the biological community.

Reviewer #3 (Remarks to the Author):

We very much appreciate the reviewer's insightful comments which have helped us improve the manuscript. Our responses are described below.

The manuscript "Combining structural modeling and deep learning to calculate the E. coli protein interactome and functional networks" describes the use of a combination of tools for the prediction of proteome-wide protein-protein interactions in E. Coli.This approach allowed to identify potential novel interactions, predicted with high likelihood.

By clustering the obtained pairings, the authors managed to identify subnetworks and describe biological process associated to them, thus allowing to predict the potential function of unannotated proteins within the

network.
I think that the described method is of great interest to annotate potential grey zones in the annotation of proteins / biological assemblies.

I was surprised not to see any code / script / repository associated with the described method. I unfortunately cannot apply it on my own system without having to write it myself from scratch.

The PrePPI pipeline, first assembled about 15 years ago, is computer cluster dependent and is challenging to distribute. It is currently being rewritten to allow for broad distribution. The ZEPPI and DSCRPIT-TT methods were published with available Github repositories (https://github.com/honig-lab/ZEPPI, https://github.com/samsledje/D-SCRIPT). We now uploaded the code that was developed in this work to take input from PrePPI, ZEPPI and TT databases and integrate them through a Bayesian model. Besides the direct model codes, we also uploaded two Jupyter notebooks for model training and testing, respectively, providing step-by-step guidelines. The Github link is https://github.com/honig-lab/BayesianModel-for-Ecoli-PPI. In the revised Methods section, we have also added more detailed descriptions explaining the details of the Bayesian model employed in this study.

Here are some remarks related to the text:

l.174: It is not clear why only SM-lr, TT-prob, TT-lr and Int-lr are evaluated against the HINT E.Coli binary dataset, but not ZEPPI-lr. Is it because it requires structural model to perform predictions?

We apologize for the confusion. All methods including ZEPPI were evaluated against HINT *E. Coli* and these results appear in the revision (Table 1A).

But because in the introduction is is mentioned that Int-lr contains prediction from ZEPPI-lr, therefore I am getting confused. I would suggest adding the reasons why ZEPPI-lr is not part of Table 1, nor in both Figure 1 and 2, or to add it. Maybe a short description of what is required by ZEPPI in the text would help? And in l.210, it is again stated that ZEPPI-lr is part of the Int-lr, making it again surprisingly confusing.

We avoided ZEPPI alone because it requires PrePPI and MSAs. But the reviewer is right that our choice was confusing and ZEPPI has now been added with a short description (Table 1-2).

l.192: AU, potentially meaning Area Under, is not defined.

Thanks. Now defined.

l.196: In figure 2, I am surprised not to see any comparisons against the HINT E.Coli dataset. How many predictions are corrected, how many are missing, how many new potential binary pairings have been found?

We have added a table (Table 1) to answer these questions.

l.207: I have the feeling that I need more explanations about the content of those 400 PPI chosen. How many of them are already reported in the literature? How many of them are new ones?

The number has decreased to 374 due to a new stage of filtering and this information can now be found in Methods: *"Starting with the 19,469 protein-protein interactions with LRs below the FPR cutoff of ≤0.001 (Table 1B) we selected PPIs for which the PrePPI SM models have at least 5 interfacial contacts among heavy atoms with a distance cutoff of < 6Å. From this set, we retained challenging cases involving pairs whose members have low local sequence identity (< 40%) and high alignment length (> 100 residues) with the corresponding chains from the PDB template complex selected by PrePPI for model evaluation. Further, to avoid overrepresentation, no protein appears more than six times in this set. Finally, from the highest-confidence PPIs matching these criteria, 374 were chosen so that half appear in databases and, thus, the other half are novel predictions. Detailed information on the final set of 374 PPIs is provided in Table S1A."*

l.214: to my understanding, I would guess that the actual sentence should be "… 80% of PDB complexes are recapitulated ABOVE this threshold", instead of "… 80% of PDB complexes are recapitulated at this threshold"?

Thanks. Corrected in revision.

l.215: I would suggest to add reasons why AF3Complex is not producing scores for the 18 entries.

We point out in the manuscript that AF3-based methods do not always produce good models and sometimes none at all. We are not aware of a detailed analysis of the cause of this failure. AF3 performs less accurately on complexes that are significantly different from the data on which it was trained. Since we have chosen cases without representative PDB homologs, we surmise this is the reason. We discuss this further in the manuscript.

l.214-215 and Table S1A: I see that there is a substantial amount of homo-dimeric complexes that are selected among the 400 PPI chosen. I would suggest to discuss this behavior and the underlying number of homo- and hetero-mers that are modelled. Is there a bias in the selection methods where homo-dimeric complexes can easily be chosen as the < 40% cutoff on sequence identity can only be met on the same chain and not on both ?

We chose a preponderance of heterodimers simply because there are many more of them in the proteome. The numbers are provided in Methods. Among the 374 PPIs modeled by AF3Complex, the average pIS is 0.63 for homodimers and 0.41 for heterodimers. Interestingly, all of the cases for which AF3Complex fails are for heterodimeric PPIs. We point this out in Results. Tables S1 and S2 are now available on Figshare https://dx.doi.org/10.6084/m9.figshare.30822977.

l.232-232: For well scored complexes, if both PrePPI (homology modelling method) and AF3Complex (machine-learning method) produce similar results, I have the feeling that there might be a bias in the use of pIS score. Indeed AF3Complex has (most probably) seen the complexes used by PrePPI to model the complex and therefore these complex are not so novel. My concerns is more related to the possibility for the overall pipeline (Int-lr into AF3Complex) to validate new / undiscovered pairings.

The reviewer is absolutely right and we have revised this section so as to remove any suggestion that the AF3 models provide validation. We now emphasize the value in comparing and using both types of models.

l.263: If I do understand well, for the remaining 73 clusters (558 - 485), not any of the proteins are annotated ? Are these big clusters ? or simply pairings of unannotated proteins ?

Among the clusters with no statistically significant functional annotation, most (65) have three or two members. While we do find annotations for many small clusters, statistical limitations become more pronounced with fewer proteins, such as lower statistical power to detect enrichment and issues with false discovery rate (FDR) correction (e.g. PMID: 39539946). The largest unannotated cluster contains 11 members, however this an extremely sparse network: Of the 11 edges, 10 are mediated by a single protein, which has only a single associated GO biological process term (polyphosphate biosynthetic process). This information is now included in the Results.

l.731: Figure 4. I would suggest to add the number of novel PPI and known PPI from curated databases, as counting the red and grey lines is hard (impossible). Possibly as percentages of known and unknown ? and reporting the total number of edges present in each cluster ?

A table enumerating the edges in the clusters in Figure 2 was added (Table S2G) and is referred to in the text. Tables S1 and S2 are now available on Figshare https://dx.doi.org/10.6084/m9.figshare.30822977.

l.302: AF3Complex suddenly becomes AlphaF3Complex ?

Thanks. Corrected in the revision.

l.327-376: Discussion: In my opinion, this resembles more a conclusion rather than an actual discussion. Therefore the discussion is missing from the current version of the paper.

After rereading the discussion, we totally agree. It has been rewritten and we hope the reviewer finds it more interesting.

l. 394: It is unclear why 4 million predictions cannot be made by PrePPI.

As now pointed out in the paper, the problem is that a good template cannot always be found. The problem is less severe for larger proteomes – especially human.

l.408-409: The ZEPPI-lr is trained on the HINT E.Coli but not human, making it the only method trained on E.Coli and also used to predict E.Coli partners ? Why would it be ? And not trained on Human HINT instead ?

This was an error in text that wasn't caught by us. All methods including ZEPPI were trained on human. For clarity, we have rephrased related sentence to *"This is accomplished by training ZEPPI Z-scores on HINT human dataset and then using the trained model on E. coli."*.

l.430: "During training, …": it is not clear here what is the training set and the loss function use for the Bayesian model.

In the revised Methods section, we added detailed descriptions for the training process of the Bayesian model. Since we used the naïve-Bayesian model, which is a general, purely statistics-based machine-learning framework, no loss function was applied here. Related details can also be found in our

uploaded model code and tutorial notebooks (https://github.com/honig-lab/BayesianModel-for-Ecoli-PPI).

REVIEWERS' COMMENTS

Reviewer #1 (Remarks to the Author):

The revised manuscript in its current form is suitable for publication.

Reviewer #2 (Remarks to the Author):

The revised paper provides fast pipeline to identify protein complexes at genomic scale demonstrating its application on Ecoli-PPI, which is current frontier in molecular systems biology. It uses template based pipeline PrePPI, assessor of interface quality ZePPI, and STRING trained sequence based prediction and uses combined model to predict interactions. The model is fast for use at proteomic scale prediction . It gives comparable results to AF based models, and also can be used in conjunction. The authors demonstrate that clustering proteins based on predicted PPIs can provide functional annotation. Overall the authors present blueprint for fast analysis of organism PPI networks and provide predictions for EColi. The papers is well written, methodology is well described.

Reviewer #2 (Remarks on code availability):

The instructions appear adequate and there are test examples related to the paper.

Reviewer #3 (Remarks to the Author):

I appreciated the point by point responses to the reviewers and believe that the manuscript has accordingly improved in the right direction.
Nevertheless, I am allowing to add several issues and remarks that I could find that could optimize once more that text:

l.199: Small typo where “At an FPR…” should be (in my opinion) “At a FPR…”

We have kept “At an FPR..”

l.200: same

We have kept “At an FPR..”

l.221: I do not fully agree with the sentence “Thus, we regard the 83 protein complexes as not being predicted by AF3Complex.”. In my opinion, the predictions are made, but not trustworthy, “thus, we considered the 83 protein complexes poorly predicted by AF3Complexe as false positive” ?

We have removed this ambiguous sentence.

l. 207: General comments about the “AF3Complex models of predicted PPIs”, in relation to the following part “Clustered PPIs yield functional subnetworks”. I have the feeling that because of the stringent selection of the 374 diverse binary PPIs, some of the failures in modelling could come for the fact that predicted PPIs are belonging to higher order macro-molecular complexes (or with a different stoichiometry). Have you considered to model not only the predicted pair, but its full subnetwork ? And, in the same thought, are those 83 poorly predicted binary complexes coming from predictions made by TTlr only ?

The current manuscript is limited to the consideration of binary PPIs. The INT^{LR} predictions calculated by AF3Complex were chosen as described in the text without consideration of the clustering analysis. The clusters were annotated with AF3Complex predictions after the analysis and these models are highlighted only to provide an additional layer of mechanistic information.

l.283: small typo, missing the verb in "however this an extremely sparse network" that should probably become "however this is an extremely sparse network"

Fixed

l.345: maybe small typo in "The three methods leverage differ data sources." that should probably become "The three methods leverage different data sources."

Fixed.

l.348: small typo, the parenthesis should become a coma

Fixed

l.349-350: I would modify "…yields the best results for AUROC and AUPR" to "…yields the best performances in terms of AUROC and AUPR"
l.377: et al. should be in italic.

Fixed

l.378: I feel strange the "…is to report…". Shouldn't it be "…reports…" or "… is reporting…" ?
??
l.381: et al. should be in italic.

Fixed

Table 1: I would add the number of undetected PPI from HINT E.Coli in all cases, even though this is partially present in the PR-AUC, and in the text (l.192 to 196)

Table 1. has now been broken up into two tables (1 and 2) in part to address this suggestion and in part to address the editorial instructions not to label tables as 1A and 1B.

Table 2: I feel there is a mistake in the caption that mention TTprob while the table contains the values for TTlr. Also, I would display only half of the table, as it is a symmetric one, and therefore add some unnecessary noise. Finally, why not also showing the values at FPR<000.1 ?

All information is now provided.

Reviewer #3 (Remarks on code availability):

The README.md is missing the python3 version required.

"Requires Python 3.9 or later" was added to README.md.

It is missing instructions on how to write the UniprotID_pair. Is it coma separated ? is it underscore separated ?

What is the filename that should be given to this file ?

This information is now provided.

In both `Train_on_human.ipynb` and `Train_on_human.ipynb, `localpath_prefix` variable must be changed. I would suggest to add a comment before to make sure the user will do it.
Same for `filetoread\d?`: the hardcoded filepaths will likely not match anything present on the user computer and nothing present in the repo.

Following the reviewer's suggestion, we have added comments clarifying the `localpath_prefix` variable.

There is no instructions on how to download the HINT and STRING databases.

Instructions have been added to README.md

Responding to editor's instructions, we uploaded the PrePPI, ZEPPI, and D-SCRIPT (TT) predictions for Human and E. coli. and the curated experimental databases for human to Figshare.

I could not find anything related to the Cytoscape analysis.

This has been added to Methods.

While the notebooks are showing the basics of the analysis, reproducing it is not possible, as many files cannot be found. Maybe a storage space such as zenodo, or making the OneDrive open could be a solution ?

A significantly improved code package is available via GitHub and integrated with Zenodo.
